# Supplementary material for: An RNA Interference (RNAi) Toolkit and Its Utility for Functional Genetic Analysis of Leishmania (Viannia)
Source: Genes (Basel). 2022 Dec 28;14(1):93. doi: 10.3390/genes14010093 (PMC9858808; doi:10.3390/genes14010093)

Nov 11 2022

Supporting information for

An RNA interference (RNAi) toolkit and its utility for functional genetic analysis of *Leishmania (Viannia)*; Lon-Fye Lye, Katherine L. Owens, Soojin Jang, Joseph E. Marcus, Erin A. Brettmann, and Stephen M. Beverley

---

Supplemental Figure S1.

Western Blot analysis of *HGPRT*-StL transfectants.

HGPRT StL constructs with varying stem lengths (500, 1005, 679, 499 bp) were transfected into *L. braziliensis* and clonal lines recovered. Two of each were subjected to Western blotting with anti-HGPRT and anti-H2A, and the relative expression was calculated and normalized to WT. M, molecular weight marker; WT, *Lbr*.

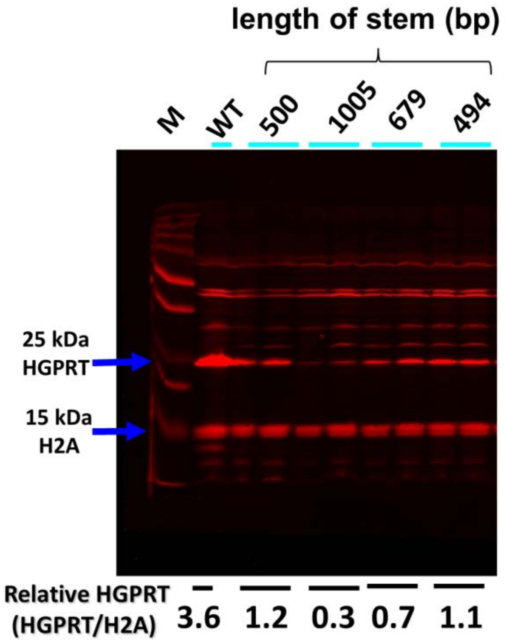

Supplement: Supplementary file 1 [file genes-14-00093-s001.zip › Supplementary Figure S1 Nov 11 2022 FINAL.pdf]
