# Supplementary material for: An RNA Interference (RNAi) Toolkit and Its Utility for Functional Genetic Analysis of Leishmania (Viannia)
Source: Genes (Basel). 2022 Dec 28;14(1):93. doi: 10.3390/genes14010093 (PMC9858808; doi:10.3390/genes14010093)
Supplement: Supplementary file 1 [file genes-14-00093-s001.zip › Supplementary File S1 - sequence of pIR1HYG-GW FINAL.pdf]

Nov 11 2022

Supporting information for

An RNA interference (RNAi) toolkit and its utility for functional genetic analysis of *Leishmania* (*Viannia*); Lon-Fye Lye, Katherine L. Owens, Soojin Jang, Joseph E. Marcus, Erin A. Brettmann, and Stephen M. Beverley

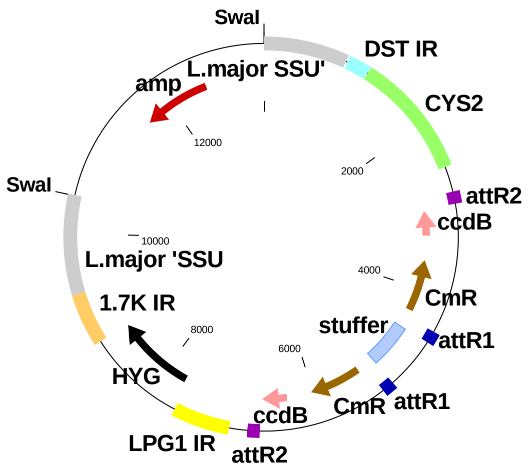

Supplemental File S1. Sequence of a representative pIR-GW vector, pIR1HYG-GW

LOCUS pIR1HYG-GW(B) 13288 bp DNA CIRCULAR SYN 10-NOV-2022
COMMENT Beverley lab strain B6544
FEATURES Location/Qualifiers
misc_feature 1..913
/gene="L.major SSU'"
misc_feature 942..1179
/gene="DST IR"
misc_feature 1195..2532
/gene="CYS2"
misc_feature complement (2824..2948)
/gene="attR2"
CDS complement (2989..3294)
/gene="ccdB"
/product="ccdB gene for negative selection"

```
     CDS             complement (3636..4295)
                     /gene="CmR"
                     /product="Chloramphenicol resistance gene"
     misc_feature    complement (4404..4528)
                     /gene="attR1"
     CDS             4544..5108
                     /gene="PEX11-MYC stuffer"
     misc_feature    5120..5244
                     /gene="attR1"
                     /product="attR1 recombination site"
     CDS             5353..6012
                     /gene="CmR"
                     /product="Chloramphenicol resistance gene"
     CDS             6354..6659
                     /gene="ccdB"
                     /product="ccdB gene for negative selection"
     misc_feature    6700..6824
                     /gene="attR2"
     misc_feature    7036..7654
                     /gene="LPG1 IR"
                     /product="5'LPG1 Intergenic Region from L. don"
     CDS             7717..8742
                     /gene="HYG"
                     /product="hygromycin resistance gene
     misc_feature    8773..9340
                     /gene="1.7K IR"
     misc_feature    9347..10426
                     /gene="L.major 'SSU"
     CDS             complement (11643..12503)
                     /gene="amp"
                     /product="ampicillin resistance"
BASE COUNT     3074 a   3470 c   3359 g   3385 t
ORIGIN
        1 aaattggata acttggcgaa acgccaagct aatacatgaa ccaaccgggt gttctccact
       61 ccagacggtg ggcaaccatc gtcgtgagac gcccagcgaa tgaatgacag taaaaccaat
      121 gccttcactg gcagtaacac ccagcagtgt tgactcaatt cattccgtgc gaaagccggc
      181 ttgttccggc gtcttttgac gaacaactgc cctatcagct ggtgatggcc gtgtagtgga
      241 ctgccatggc gttgacggga gcgggggatt agggttcgat tccggagagg gagcctgaga
      301 aatagctacc acttctacgg agggcagcag gcgcgcaaat tgcccaatgt caaaacaaaa
      361 cgatgaggca gcgaaaagaa atagagttgt cagtccattt ggattgtcat ttcaatgggg
      421 gatatttaaa cccatccaat atcgagtaac aattggagga caagtctggt gccagcaccc
      481 gcggtaattc cagctccaaa agcgtatatt aatgctgttg ctgttaaagg gttcgtagtt
      541 gaactgtggg ctgtgcaggt ttgttcctgg tcgtcccgtc catgtcggat ttggtgaccc
      601 aggcccttgc agcccgtgaa cattcaaaga aacaagaaac acgggagtgg ttcctttcct
      661 gatttacgca tgtcatgcat gccagggggc gtccgtgatt ttttactgtg actaaagaag
      721 cgtgactaaa gcagtcattt gacttgaatt agaaagcatg ggataacaaa ggagcagcct
```

781 ctaggctacc gtttcggctt ttgttggttt taaaggtcta ttggagatta tggagctgtg
841 cgacaagtgc tttcccatcg caacttcggt tcggtgtgtg gcgcctttga ggggtttagt
901 gcgtccggtg cgatagggag accacaacgg tttccctcta gtgcgtgaag ggttaccgca
961 acgatgcgca atggactccc ccgctttcca tttcgtcacc ttccgcctct ctctctctct
1021 ctctcaccat ctacgcgtgc acatcatcaa ctgtctcttg tcggtgctca ccaccctcaa
1081 ccacccctca ctttcaaggc ttcccgaacg cacacaaaag gcgtgaaaac cgctcgcgtg
1141 tgttgagccg tccaccgtag ccctccccct gtccccgggg gatccactag ttctagagga
1201 tcggaggtgt gtgtgccctt gtgtgctgtg tgtgggtgga cgcagcgatg cccggcgcgt
1261 gtgggcacct ccttgggtgc gcgcccgccg tggcagctgc gcgtgcgtgc gagatgtgag
1321 gcagaggaag aggaaggcga tgcgggcgac acgcagaggt gcggcggacg taggggggaa
1381 atggacgagc aggcgcgctg tgaatcggag ctgcggcacc acccaagtcg tggtgccccg
1441 cgaatggctg ttctgccgcc ctcgcttcac gcctccccct cccctcgcgt gccctcgcgt
1501 ggcctccctt gttatccctc tctctcgcac gcacacggat acgcgagccc gctattctgc
1561 cttcgtctgg ctctttgtat tctgcttgct tcttcagcac acttgtgtgc tgtgcgttca
1621 gcgatatctt ccactacttt gttttctcct ccccctcggg aggtgcttcg cttgtgcttt
1681 gacggtggtg cgtggctgct gggtcatgtg ccgggcgtgc gcgcctccgc cgcctccctg
1741 cagcttgtgg gtctggctgc gttcgcaccg cgctcgcgtg catgcacatg cctgcactgc
1801 gtcgggaacg acttccgggc gcgttggccc cccgcctctg cagccacggt ctgtttattg
1861 attgtgcttg cttcatcggc tcttctctgc gcgcgtgcgt gcgtgcgtgt gcgtgtccgt
1921 gcgtatgcgt gaggcgcaac ggtccccaga gcaaggcatg tcgaggggaa cactatagac
1981 gcatgtgtac gtgtacacga tgtgtatacg tatacgtgta ccgaatggtg cgtgcgcgtg
2041 tgcagcattg ccgtgacggc atgtacgaag cgctgcagtg ggatggaccc tgtgcgcgtg
2101 ccggagaggt agtgtcgcgt gtgggtgcgg agtgatggag gctagggggc ttacgagcac
2161 cgtcgctttt cccccgatgg cggctggcac gcagcgcacg caccggggat gtgtgacgtg
2221 cgtcctgtgc gcctctccct ctccccttgt cgccggcgca tggatgcacc gctgttgtgt
2281 gaggttgccc gcacctgcgt tgttgcctgt gatgacgtcc ctccctctct tgcactctcc
2341 ccgtccccac ctgccctgca ccgtggtcga ctgctcccga cgccctgcac agactctcgt
2401 cgccaccacc agcagcagcc ctctatatac ccgccactgc cgtagcgttc gggccgtggc
2461 tctgcgtttc acttgctctc ccctcgctct gttcattgct tccttctgtt cccctcgctg
2521 cccgcgtccg gagatcagcg gaagagcgcc caatacgcaa accgcctctc cccgcgcgtt
2581 ggccgattca ttaatgcagg actaattaac cctcactaaa gggagatccg ggtcgtattg
2641 atttggcgtt acgcgaacgc gaagtccgac tctaagatgt cacggaggtt caagttacct
2701 ttagccggaa gtgctggcat tttgtccaat tgagactcgt gcaactggtc agcgaactgg
2761 tcgtagaaat cagccagtac atcacaagac tcatagatca gcttggccta gattaattaa
2821 atcaccactt tgtacaagaa agctgaacga gaaacgtaaa atgatataaa tatcaatata
2881 ttaaattaga ttttgcataa aaaacagact acataatgct gtaaaacaca acatatccag
2941 tcactatggt cgacctgcag actggctgtg tataagggag cctgacattt atattcccca
3001 gaacatcagg ttaatggcgt ttttgatgtc attttcgcgg tggctgagat cagccacttc
3061 ttccccgata acggagaccg gcacactggc catatcggtg gtcatcatgc gccagctttc
3121 atccccgata tgcaccaccg ggtaaagttc acgggagact ttatctgaca gcagacgtgc
3181 actggccagg gggatcacca tccgtcgccc gggcgtgtca ataatatcac tctgtacatc
3241 cacaaacaga cgataacggc tctctctttt ataggtgtaa accttaaact gcatttcacc
3301 agcccctgtt ctcgtcagca aaagagccgt tcatttcaat aaaccgggcg acctcagcca
3361 tcccttcctg attttccgct ttccagcgtt cggcacgcag acgacgggct tcattctgca
3421 tggttgtgct taccagaccg gagatattga catcatatat gccttgagca actgatagct
3481 gtcgctgtca actgtcactg taatacgctg cttcatagca tacctctttt tgacatactt

3541 cgggtataca tatcagtata tattcttata ccgcaaaaat cagcgcgcaa atacgcatac
3601 tgttatctgg cttttagtaa gccggatcca cgcgtttacg ccccgccctg ccactcatcg
3661 cagtactgtt gtaattcatt aagcattctg ccgacatgga agccatcaca aacggcatga
3721 tgaacctgaa tcgccagcgg catcagcacc ttgtcgcctt gcgtataata tttgcccatg
3781 gtgaaaacgg gggcgaagaa gttgtccata ttggccacgt ttaaatcaaa actggtgaaa
3841 ctcacccagg gattggctga gacgaaaaac atattctcaa taaacccttt agggaaatag
3901 gccaggtttt caccgtaaca cgccacatct tgcgaatata tgtgtagaaa ctgccggaaa
3961 tcgtcgtggt attcactcca gagcgatgaa aacgtttcag tttgctcatg gaaaacggtg
4021 taacaagggt gaacactatc ccatatcacc agctcaccgt ctttcattgc catacggaat
4081 tccggatgag cattcatcag gcgggcaaga atgtgaataa aggccggata aaacttgtgc
4141 ttatttttct ttacggtctt taaaaaggcc gtaatatcca gctgaacggt ctggttatag
4201 gtacattgag caactgactg aaatgcctca aaatgttctt tacgatgcca ttgggatata
4261 tcaacggtgg tatatccagt gatttttttc tccattttag cttccttagc tcctgaaaat
4321 ctcgacggat cctaactcaa aatccacaca ttatacgagc cggaagcata aagtgtaaag
4381 cctggggtgc ctaatgcggc cgccaatatg actggatatg ttgtgtttta cagtattatg
4441 tagtctgttt tttatgcaaa atctaattta atatattgat atttatatca ttttacgttt
4501 ctcgttcagc tttttgtac aaacttgtga tagatcttct aggaagatcc tcctcggata
4561 ttaacttctg ctcaccgttg aggtcttcct cactgatcaa tttctgttct ccattcaagt
4621 cctcttcaga aatgagcttt tgctcagagc ggcggatccg ggcttgagtc gtagccaggg
4681 acagattagg gtgagaatag acagaatgga ggatatgagg ccacacagcc ccacaatccc
4741 agggccacaa cgccagaggc ctagtttgtc cagtgggatg aaaaggtcac aggcatttct
4801 gagcacatcc agcaagagag ggggatgacc tcgaaggaca cgagccagta gcaggattcg
4861 gagccgaaac ttcaaagcca gttgtggcag acttcctcct ggagtccctg gtcctccagg
4921 tcccacagtt tctactcctc ctgagactcc tactccagaa accttcattc gcctgctgta
4981 agctgaagtc tcttgttcca tcaataggcg aatctcataa gcatcacggc tcagattcat
5041 aatgagggaa aacagatagt atctgaatga acgctgtgcc cacttttcct ggtccactcg
5101 gggagctaga agatctatca caagtttgta caaaaaagct gaacgagaaa cgtaaaatga
5161 tataaatatc aatatattaa attagatttt gcataaaaaa cagactacat aatactgtaa
5221 aacacaacat atccagtcat attggcggcc gcattaggca ccccaggctt tacactttat
5281 gcttccggct cgtataatgt gtggattttg agttaggatc cgtcgagatt ttcaggagct
5341 aaggaagcta aaatggagaa aaaaatcact ggatatacca ccgttgatat atcccaatgg
5401 catcgtaaag aacattttga ggcatttcag tcagttgctc aatgtaccta taaccagacc
5461 gttcagctgg atattacggc ctttttaaag accgtaaaga aaaataagca caagttttat
5521 ccggccttta ttcacattct tgcccgcctg atgaatgctc atccggaatt ccgtatggca
5581 atgaaagacg gtgagctggt gatatgggat agtgttcacc cttgttacac cgttttccat
5641 gagcaaactg aaacgttttc atcgctctgg agtgaatacc acgacgattt ccggcagttt
5701 ctacacatat attcgcaaga tgtggcgtgt tacggtgaaa acctggccta tttccctaaa
5761 gggtttattg agaatatgtt tttcgtctca gccaatccct gggtgagttt caccagtttt
5821 gatttaaacg tggccaatat ggacaacttc ttcgcccccg ttttcaccat gggcaaatat
5881 tatacgcaag gcgacaaggt gctgatgccg ctggcgattc aggttcatca tgccgtttgt
5941 gatggcttcc atgtcggcag aatgcttaat gaattacaac agtactgcga tgagtggcag
6001 ggcggggcgt aaacgcgtgg atccggctta ctaaaagcca gataacagta tgcgtatttg
6061 cgcgctgatt tttgcggtat aagaatatat actgatatgt atacccgaag tatgtcaaaa
6121 agaggtatgc tatgaagcag cgtattacag tgacagttga cagcgacagc tatcagttgc
6181 tcaaggcata tatgatgtca atatctccgg tctggtaagc acaaccatgc agaatgaagc
6241 ccgtcgtctg cgtgccgaac gctggaaagc ggaaaatcag gaagggatgg ctgaggtcgc

6301 ccggtttatt gaaatgaacg gctcttttgc tgacgagaac aggggctggt gaaatgcagt
6361 ttaaggttta cacctataaa agagagagcc gttatcgtct gtttgtggat gtacagagtg
6421 atattattga cacgcccggg cgacggatgg tgatccccct ggccagtgca cgtctgctgt
6481 cagataaagt ctcccgtgaa ctttacccgg tggtgcatat cggggatgaa agctggcgca
6541 tgatgaccac cgatatggcc agtgtgccgg tctccgttat cggggaagaa gtggctgatc
6601 tcagccaccg cgaaaatgac atcaaaaacg ccattaacct gatgttctgg ggaatataaa
6661 tgtcaggctc ccttatacac agccagtctg caggtcgacc atagtgactg gatatgttgt
6721 gttttacagc attatgtagt ctgtttttta tgcaaaatct aatttaatat attgatattt
6781 atatcatttt acgtttctcg ttcagctttc ttgtacaaag tggtgattta attaatctag
6841 cgaccagatc tatgagtctt gtgatgtact ggctgatttc tacgaccagt tcgctgacca
6901 gttgcacgag tctcaattgg acaaaatgcc agcacttccg gctaaaggta acttgaacct
6961 ccgtgacatc ttagagtcgg acttcgcgtt cgcgtaacgc caaatcaata cgacccggat
7021 ctccctttag tgagggttaa ttagtcctgc attaatgaat cggccaacgc gcggggagag
7081 gcggtttgcg tattgggcgc tcttccgcta ctcgggtgtc gcacacactg taaaacgccc
7141 ccgccggctc tgtcacgcaa gaaacgagag caaaaagacc ggtagactat atcacgcaca
7201 atcaccgcgt gtgcgtctcc ctgggtgaag acacccatcg cacccttcga cagccgccct
7261 tatgcctatt caccgtctgt agaacacaca agaggaatag cccggtgccg cgtgcaagac
7321 tgcggcttct gcacgcacta tgctcgtttc cgcctctctc tctttgtgcg cgtgtgtgtg
7381 tgtgtgtcgg agtggccctc ccgttacgtc ttttgggggt gggtgatagc ggcagatgct
7441 gcttcgacct tgtgcgccgc accggtgccg ttggctacac tgcggaaggc aacacagaac
7501 acaccctgtg ccatttcttc ttttttttt gctttcaccc accttttccc cgtgcttccc
7561 catctttccc cctctttccc taacgtacat tgcacctctc cttatcgtgc agtcacacgc
7621 taccactcaa cgctccctgc aacactggag tgagtcgcta gaaataattt tgtttaactt
7681 taagaaggag atatacatag tgaccggatc tccaccatga aaaagcctga actcaccgcg
7741 acgtctgtcg agaagtttct gatcgaaaag ttcgacagcg tctccgacct gatgcagctc
7801 tcggagggcg aagaatctcg tgctttcagc ttcgatgtag gagggcgtgg atatgtcctg
7861 cgggtaaata gctgcgccga tggtttctac aaagatcgtt atgtttatcg gcactttgca
7921 tcggccgcgc tcccgattcc ggaagtgctc gacattgggg aattcagcga gagcctgacc
7981 tattgcatct cccgccgtgc acagggtgtc acgttgcaag acctgcctga aaccgaactg
8041 cccgctgttc tgcagccggt cgcggaggcc atggatgcga tcgctgcggc cgatcttagc
8101 cagacgagcg ggttcggccc attcggaccg caaggaatcg gtcaatacac tacatggcgt
8161 gatttcatat gcgcgattgc tgatccccat gtgtatcact ggcaaactgt gatggacgac
8221 accgtcagtg cgtccgtcgc gcaggctctc gatgagctga tgctttgggc cgaggactgc
8281 cccgaagtcc ggcacctcgt gcacgcggat ttcggctcca acaatgtcct gacggacaat
8341 ggccgcataa cagcggtcat tgactggagc gaggcgatgt tcggggattc ccaatacgag
8401 gtcgccaaca tcttcttctg gaggccgtgg ttggcttgta tggagcagca gacgcgctac
8461 ttcgagcgga ggcatccgga gcttgcagga tcgccgcggc tccgggcgta tatgctccgc
8521 attggtcttg accaactcta tcagagcttg gttgacggca atttcgatga tgcagcttgg
8581 gcgcagggtc gatgcgacgc aatcgtccga tccggagccg ggactgtcgg gcgtacacaa
8641 atcgcccgca gaagcgcggc cgtctggacc gatggctgtg tagaagtact cgccgatagt
8701 ggaaaccgac gccccagcac tcgtccgagg gcaaaggaat agcctagttc tagtctaggg
8761 ccgaatcaga tcctcgtgtg agcgttcgcg gaatcggtcg ctcgtgttta tgcccgtctt
8821 ggtgttgtgc tcgcaaggcg gtgcagcagg ataccgtcgc cctcctctct ccttgcttct
8881 ctgttcttca attcgcgatc tcacagaggc cggctgtgca cgcccttcct caccctcctt
8941 ttcccacctc tcggccaccg gtcggctccg ttccgtctgc cgtcgagaag ggacgggcat
9001 gtgcagctcc tccctttctc tcgcgcgcgc atcttctctt gcttgtggca ctcacgctca

9061 tgcgtcaagg cggccccacg cgagcccctg cgctcccttc cctcttgcgc atccgtagcc
9121 ggactggtcg atgcgcaagg ccggcatgaa ggagcgcgtg ccctcaagag cggactatca
9181 tgccctacgt gggccacgca gcgatgaggc cggcttcgcg gagatgcgtc acgcacgtgc
9241 cagatgatgc cgtacgcctt ccttgacttg cgccccccctc tcttcctccg tctctcactc
9301 tctctctctc acacacacac acacacacac acacacacaa agctccggtt cgtccggccg
9361 taacgccttt tcaactcacg gcctctagga atgaaggagg gtagttcggg ggagaacgta
9421 ctggggcgtc agaggtgaaa ttcttagacc gcaccaagac gaactacagc gaaggcattc
9481 ttcaaggata ccttcctcaa tcaagaacca aagtgtggag atcgaagatg attagagacc
9541 attgtagtcc acactgcaaa cgatgacacc catgaattgg ggatcttatg ggccggcctg
9601 cggcagggtt taccctgtgt cagcaccgcg cccgctttta ccaacttacg tatcttttct
9661 attcggcctt taccggccac ccacgggaat atcctcagca cgttttctgt tttttcacgc
9721 gaaagctttg aggttacagt ctcagggggg agtacgttcg caagagtgaa acttaaagaa
9781 attgacggaa tggcaccaca agacgtggag cgtgcggttt aatttgactc aacacgggga
9841 actttaccag atccggacag gatgaggatt gacagattga gtgttctttc tcgattccct
9901 gaatggtggt gcatggccgc ttttggtcgg tggagtgatt tgtttggttg attccgtcaa
9961 cggacgagat ccaagctgcc cagtagaatt cagaattgcc catagaatag caaactcatc
10021 ggcgggtttt acccaacggt gggccgcatt cggtcgaatt cttctctgcg ggattccttt
10081 gtaattgcac aaggtgaaat tttgggcaac agcaggtctg tgatgctcct caatgttctg
10141 ggcgacacgc gcactacaat gtcagtgaga acaagaaaaa cgacttttgt cgaacctact
10201 tgatcaaaag agtggggaaa ccccggaatc acatagaccc acttgggacc gaggattgca
10261 attattggtc gcgcaacgag gaatgtctcg taggcgcagc tcatcaaact gtgccgatta
10321 cgtccctgcc atttgtacac accgcccgtc gttgtttccg atgatggtgc aatacaggtg
10381 atcggacagg cggtgtttta tccgcccgaa agttcaccga tatttaaatc cagcttttgt
10441 tccctttagt gagggttaat tgcgcgcttg gcgtaatcat ggtcatagct gtttcctgtg
10501 tgaaattgtt atccgctcac aattccacac aacatacgag ccggaagcat aaagtgtaaa
10561 gcctggggtg cctaatgagt gagctaactc acattaattg cgttgcgctc actgcccgct
10621 ttccagtcgg gaaacctgtc gtgccagctg cattaatgaa tcggccaacg cgcggggaga
10681 ggcggtttgc gtattgggcg ctcttccgct tcctcgctca ctgactcgct gcgctcggtc
10741 gttcggctgc ggcgagcggt atcagctcac tcaaaggcgg taatacggtt atccacagaa
10801 tcaggggata acgcaggaaa gaacatgtga gcaaaaggcc agcaaaaggc caggaaccgt
10861 aaaaaggccg cgttgctggc gtttttccat aggctccgcc cccctgacga gcatcacaaa
10921 aatcgacgct caagtcagag gtggcgaaac ccgacaggac tataaagata ccaggcgttt
10981 ccccctggaa gctccctcgt gcgctctcct gttccgaccc tgccgcttac cggatacctg
11041 tccgcctttc tcccttcggg aagcgtggcg ctttctcata gctcacgctg taggtatctc
11101 agttcggtgt aggtcgttcg ctccaagctg ggctgtgtgc acgaaccccc cgttcagccc
11161 gaccgctgcg ccttatccgg taactatcgt cttgagtcca acccggtaag acacgactta
11221 tcgccactgg cagcagccac tggtaacagg attagcagag cgaggtatgt aggcggtgct
11281 acagagttct tgaagtggtg gcctaactac ggctacacta gaaggacagt atttggtatc
11341 tgcgctctgc tgaagccagt taccttcgga aaaagagttg gtagctcttg atccggcaaa
11401 caaaccaccg ctggtagcgg tggttttttt gtttgcaagc agcagattac gcgcagaaaa
11461 aaaggatctc aagaagatcc tttgatcttt tctacggggt ctgacgctca gtggaacgaa
11521 aactcacgtt aagggatttt ggtcatgaga ttatcaaaaa ggatcttcac ctagatcctt
11581 ttaaattaaa aatgaagttt taaatcaatc taaagtatat atgagtaaac ttggtctgac
11641 agttaccaat gcttaatcag tgaggcacct atctcagcga tctgtctatt tcgttcatcc
11701 atagttgcct gactccccgt cgtgtagata actacgatac gggagggctt accatctggc
11761 cccagtgctg caatgatacc gcgagaccca cgctcaccgg ctccagattt atcagcaata

11821 aaccagccag ccggaagggc cgagcgcaga agtggtcctg caactttatc cgcctccatc
11881 cagtctatta attgttgccg ggaagctaga gtaagtagtt cgccagttaa tagtttgcgc
11941 aacgttgttg ccattgctac aggcatcgtg gtgtcacgct cgtcgtttgg tatggcttca
12001 ttcagctccg gttcccaacg atcaaggcga gttacatgat cccccatgtt gtgcaaaaaa
12061 gcggttagct ccttcggtcc tccgatcgtt gtcagaagta agttggccgc agtgttatca
12121 ctcatggtta tggcagcact gcataattct cttactgtca tgccatccgt aagatgcttt
12181 tctgtgactg gtgagtactc aaccaagtca ttctgagaat agtgtatgcg gcgaccgagt
12241 tgctcttgcc cggcgtcaat acgggataat accgcgccac atagcagaac tttaaaagtg
12301 ctcatcattg gaaaacgttc ttcggggcga aaactctcaa ggatcttacc gctgttgaga
12361 tccagttcga tgtaacccac tcgtgcaccc aactgatctt cagcatcttt tactttcacc
12421 agcgtttctg ggtgagcaaa aacaggaagg caaaatgccg caaaaaaggg aataagggcg
12481 acacggaaat gttgaatact catactcttc ctttttcaat attattgaag catttatcag
12541 ggttattgtc tcatgagcgg atacatattt gaatgtattt agaaaaataa acaaataggg
12601 gttccgcgca catttccccg aaaagtgcca cctgacgcgc cctgtagcgg cgcattaagc
12661 gcggcgggtg tggtggttac gcgcagcgtg accgctacac ttgccagcgc cctagcgccc
12721 gctcctttcg ctttcttccc ttcctttctc gccacgttcg ccggctttcc ccgtcaagct
12781 ctaaatcggg ggctcccttt agggttccga tttagtgctt tacggcacct cgaccccaaa
12841 aaacttgatt agggtgatgg ttcacgtagt gggccatcgc cctgatagac ggtttttcgc
12901 cctttgacgt tggagtccac gttctttaat agtggactct tgttccaaac tggaacaaca
12961 ctcaacccta tctcggtcta ttcttttgat ttataaggga ttttgccgat ttcggcctat
13021 tggttaaaaa atgagctgat ttaacaaaaa tttaacgcga attttaacaa aatattaacg
13081 cttacaattt ccattcgcca ttcaggctgc gcaactgttg ggaagggcga tcggtgcggg
13141 cctcttcgct attacgccag ctggcgaaag ggggatgtgc tgcaaggcga ttaagttggg
13201 taacgccagg gttttcccag tcacgacgtt gtaaaacgac ggccagtgag cgcgcgtaat
13261 acgactcact atagggcgaa ttggattt
//
